# Dogs’ expectations about occlusion events: from expectancy violation to exploration

**DOI:** 10.1098/rspb.2023.0696

**Published:** 2023-07-26

**Authors:** Christoph J. Völter, Ana Tomašić, Laura Nipperdey, Ludwig Huber

**Affiliations:** ^1^ Comparative Cognition, Messerli Research Institute, University of Veterinary Medicine Vienna, Medical University of Vienna and University of Vienna, 1210 Vienna, Austria; ^2^ Faculty of Veterinary Medicine, University of Leipzig, 04103 Leipzig, Germany

**Keywords:** canine cognition, physical cognition, information seeking, violation of expectation, eye tracking, pupillometry

## Abstract

Previous research on human infants has shown that violations of basic physical regularities can stimulate exploration, which may represent a type of hypothesis testing aimed at acquiring knowledge about new causal relationships. In this study, we examined whether a similar connection between expectancy violation and exploration exists in nonhuman animals. Specifically, we investigated how dogs react to expectancy violations in the context of occlusion events. Throughout three experiments, dogs exhibited longer looking times at expectancy-inconsistent events than at consistent ones. This finding was further supported by pupil size analyses in the first two eye-tracking experiments. Our results suggest that dogs expect objects to reappear when they are not obstructed by a screen and consider the size of the occluding screen in relation to the occluded object. In Experiment 3, expectancy violations increased the dogs' exploration of the target object, similar to the findings with human infants. We conclude that expectancy violations can provide learning opportunities for nonhuman animals as well.

## 1. Introduction

Knowledge about the physical world is fundamental for many animals, for instance, to navigate, locate and extract food resources, and to track conspecifics, predators or prey. Research has shown that human infants have expectations about their environment that are rooted in their core knowledge (a set of fundamental, innate knowledge systems [1,2]). These core expectations concern, among other things, object properties and mechanical interactions between objects. Expectancy violations of these core expectations enhance exploration in infants. In a seminal study, Stahl & Feigenson [3] presented 11-month-old infants with demonstrations in which an object would either violate expectations about support, solidity and continuity principles (e.g. an object being hidden behind one opaque screen reappearing behind another, spatially separated screen) or not. Following these demonstrations, infants were allowed to interact with the involved familiar object among other, new objects. The results showed that, after seeing events that violated their expectations, infants explored the involved object more than after a matched control event that was consistent with these physical regularities. Following the expectancy violation, infants were also more likely to learn about a new property of the involved object. Intriguingly, even the way the infants explored the objects seemed to be informed by the specific type of expectancy violation. In particular, infants were more likely to pound the object against the ground after having witnessed an event in which the object appeared to have moved through a solid wall (solidity violation) but they dropped the object more frequently after having seen it hovering in mid-air (support violation). The authors concluded that the infants engaged in a form of hypothesis testing. In support of this account, a follow-up study showed that the infants would explore the expectancy-violating object less when a plausible explanation was provided at the end of the demonstration (i.e. by showing that there was an unseen hole in an obstacle explaining the movement trajectory of the object [4]). Another study with 13-month-old infants provided evidence that the link between seeing something unexpected and exploration is not exclusive to core expectancy violations. Indeed, exploration could also be triggered by improbable outcomes of seemingly random draws of coloured balls from a container [5].

Nonhuman animals are well known to exhibit an orientation response and increased arousal when exposed to unusual stimuli [6]. Nevertheless, little is known about the link between expectancy violation concerning physical regularities and exploration in nonhuman animals [7]. One cannot take for granted a correlation between looking times and active exploration; examples in both the developmental and comparative literature demonstrate that increased looking times do not always result in action [e.g. 8,9].

Exploration elicited by expectancy violation is an example of specific exploration, possibly driven by epistemic curiosity. Berlyne [6] distinguished between specific and diversive exploration. Whereas diversive exploration is concerned with the qualities of the stimulus—particularly the desire for perceptual or cognitive stimulation—specific exploration relates to a particular piece of information and can serve to fill knowledge gaps. Variables that drive specific exploration include novelty, complexity, incongruity and surprisingness [6]. In this context, surprise refers to a mismatch between stimulus and expectations. In this sense, specific exploration can serve to acquire knowledge about objects or events that violate expectations. Expectancy violations concerning core knowledge are considered to be a particularly strong driver for exploration in humans [2]. Yet little is known about the question of whether violations of core expectations lead to exploration in nonhuman animals. In this study, we investigated this question in one of the most popular model species in comparative cognition [10], dogs (*Canis familiaris*).

Dogs orient (with their eyes and bodies) to changes or unusual events in their environment, which Pavlov [11] called the ‘investigatory reflex’. Novelty seems to be an important trigger for this orientation response and habituation to stimuli can decrease their orientation response. These characteristics are not specific to dogs; they are also found in other species such as rats [e.g. 12–14] or humans [e.g. 15,16]. Based on evidence from expectancy violation studies, dogs also seem to possess expectations concerning certain physical regularities such as size constancy [17] and contact causality [18], but apparently not about support events [19]. We will focus in the following on occlusion events, which are the focus of the current investigation.

Occlusion events occur when an object is temporarily or partially hidden from view by another object. Understanding when something should be visible and when not might help animals to track objects and animate beings and interact with them appropriately even when they are temporarily out of sight [20]. In general, knowledge about occlusion might allow them to distinguish absence of evidence from evidence of absence [21]. Expectancy violation studies with human infants provided evidence for incremental knowledge about occlusion events over the course of the first months of life [e.g. 22–24]. Infants progressively take into account aspects such as the shape and continuity of the occluding screen as well as the size of the screen in relation to the occluded object.

The extent to which dogs understand occlusion events and the variables they can take into account when processing occlusion events are not well understood. For example, the extent to which they can master invisible displacement tasks remains controversial [25,26; for a review, see 27]. Until recently, researchers suggested that dogs mostly rely on associative learning when solving problems involving temporal occlusion of the involved objects [e.g. 25,26]. However, evidence from a number of recent expectancy violation studies suggests that dogs have at least some expectations about object permanence and occlusion events. For example, dogs reacted with increased smelling behaviour after they had seen one food type being hidden inside a container but another food type reappearing from the container after a short delay [28]. Moreover, dogs looked for longer when objects changed their size [17,29] or colour behind a screen [29] and even when a screen occluding a food reward rotated as though it had passed through the (hidden) reward [30].

Another recent study examined whether dogs would follow a moving object when it was temporarily occluded based on the object's initial speed and whether they would show longer looking times if the ball stayed out of sight for shorter or longer durations than what could be expected based on the object's initial speed [31]. The authors found some evidence for a surprise effect in experienced dogs (especially when the object moved more slowly than expected based on its initial velocity).

All of these looking time studies, however, relied on live demonstrations and manual scoring of looking times from the videos, which can bias the results by introducing 'Clever-Hans' effects (i.e. cues given inadvertently by the experimenter; see for example [32,33]) and measurement errors. Eye tracking using standardized, video-based stimuli can serve to mitigate such potential issues. In the developmental literature, pupillometry has been suggested as a superior method to measure infants' response to expectancy violations because of its temporal sensitivity and the reliability of the psychosensory pupil dilation response [34,35]. First evidence with dogs confirms that pupillometry might indeed be a sensitive measure to analyze dogs’ reactions to surprising or emotionally arousing stimuli [18,19,36–38].

In this study, we aimed at investigating dogs' expectations about occlusion events using eye tracking and pupillometry. Therefore, in the first two experiments we presented a sample of pet dogs with realistic animations depicting two different implausible occlusion events as well as plausible control events. We recorded dogs’ gaze coordinates and pupil sizes while watching the stimuli. Finally, in the third experiment we tested whether we could replicate the looking time effect when implementing the plausible and implausible occlusion events with real objects on a small stage. More importantly, following the developmental literature [3,4], we examined whether dogs would also explore an object more following an expectancy-violating event. Based on previous expectancy violation studies [17,18,28–30], we predicted longer looking times and larger pupil sizes in response to the implausible *disappear* events than to the *reappear* control events and that the surprising *disappear* event would also enhance dogs' exploration of the involved object.

## 2. Experiments 1 and 2: Eye tracking occlusion events

### (a) Material and methods

#### (i) Subjects

We tested healthy pet dogs older than 12 months. We excluded individuals whose pupil or corneal reflection was not detected well by the eye-tracking software (e.g. dogs with an irregularly shaped or light iris) prior to the data collection. In Experiment 1 (Exp. 1), we tested 14 dogs (5 border collies, 5 mixed breeds, 2 labrador retrievers, 1 collie and 1 Australian shepherd; mean age: 30.5 months, range: 13–79 months; 8 females, 6 males). In Experiment 2 (Exp. 2), in addition to the aforementioned dogs we tested three further dogs (1 Australian shepherd, 1 small Münsterländer, 1 flat coated retriever; *N* = 17; mean age: 36.5 months, range: 14–81 months; 9 females, 8 males).

#### (ii) Stimuli

In Experiments 1 and 2, we presented the dogs with a familiarization video and two different test videos. The videos showed 3D animations created in Blender 2.8 (see https://www.blender.org/) using Blender's rigid body physics simulation. The videos had a frame rate of 100 fps (exceeding dogs' flicker-fusion rates [39]) and a duration of 3.5 s (Exp. 1) and 5.0 s (Exp. 2), respectively. However, in the eye-tracking experiments we extended presentation time of the last video frame for a total video duration of 4.5 s / 5.2 s (Exp. 1 / Exp. 2) in the familiarization trials and 13.5 s / 8.5 s (Exp. 1 / Exp. 2) in the test trials.

**Figure 1.**
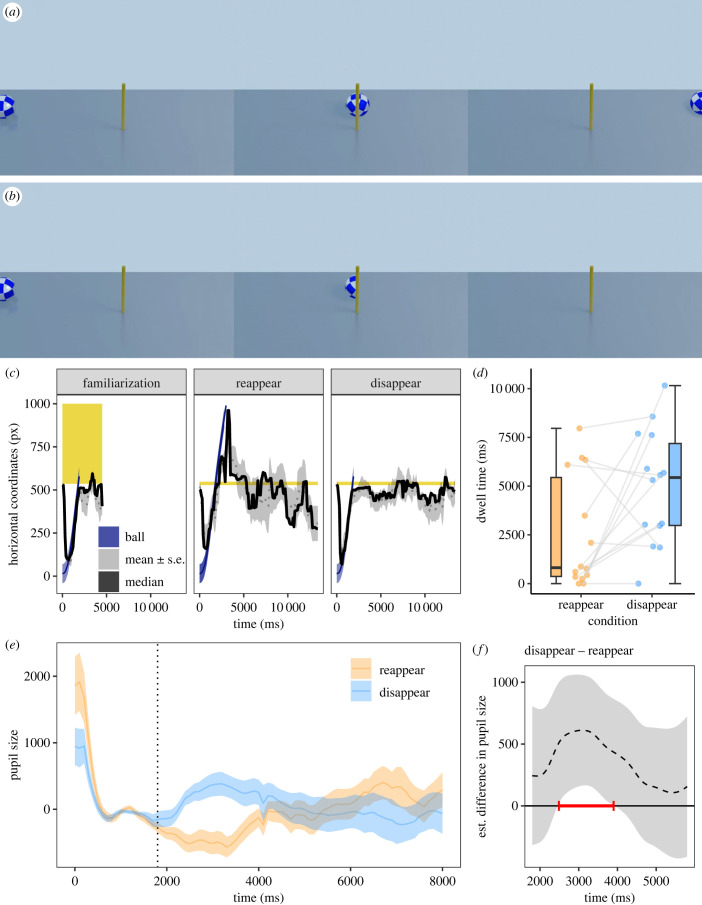
Screenshots and data visualizations of Experiment 1. Screenshots of the *reappear* condition (*a*) and the *disappear* condition (*b*) in the videos at 0, 1800 and 3121 ms (from left to right). (The videos are provided as part of the electronic supplementary material.) (*c*) Time series plot showing the dogs' median (continuous black lines) and mean horizontal gaze coordinates (± *s.e.*, dotted lines and the grey shaded areas around them; in pixels (px)) in the final familiarization trials and in the test trials. The shaded blue areas show the left and right boundaries of the ball. The yellow shaded areas indicate the area of the occluder/pole. (*d*) Dot and box plot showing the dogs’ looking times to the areas of interest around the pole at the end of the video. The dots represent the mean individual values. (*e*) Time series plot showing dogs' pupil size (in arbitrary units and baseline corrected). The blue and orange lines show the mean pupil size (± *s.e.*, shaded area around the line) in the *disappear* and *reappear* conditions, respectively. The dashed vertical line (at 1800 ms) indicates the time when the centre of the ball was behind the pole. (*f* ) Difference curve derived from a generalized additive mixed model (GAMM01) (in arbitrary units). The dashed line shows the estimated difference between the *disappear* and *reappear* test condition and the shaded area shows the pointwise 95% confidence interval. The period during which the conditions differ significantly is highlighted in red.

In Experiment 1, the familiarization video showed a blue-silver patterned ball rolling along a grey surface. A yellow rectangle occluded the right half of the screen. The ball started on the left side of the screen and rolled behind the opaque yellow wall. In the test videos, a slim yellow pole in the centre of the screen replaced the yellow wall. In both test videos, the ball rolled as before from left to right. In the *reappear* condition (figure 1*a*), the ball rolled behind the pole, reappeared on the other side, rolled further to the right edge of the screen and moved out of view. In the *disappear* condition (figure 1*b*), the ball disappeared behind the pole.

**Figure 2.**
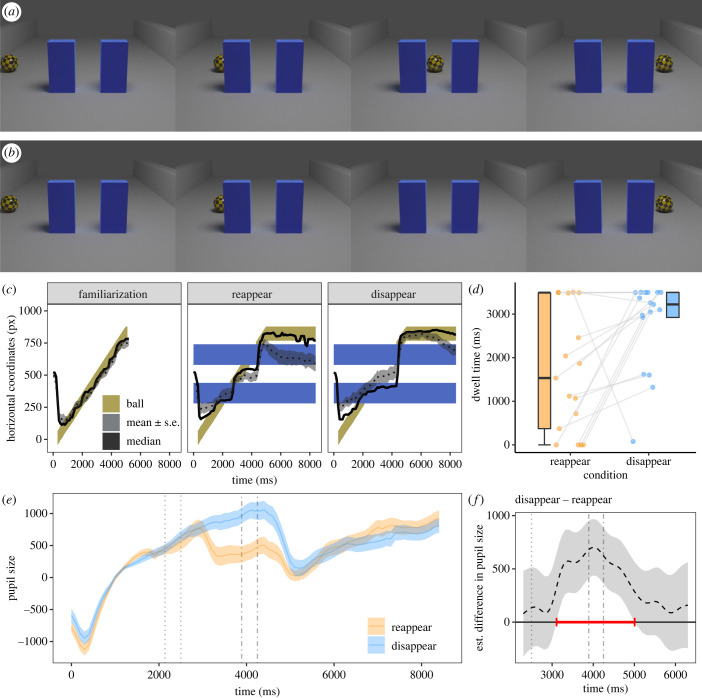
Screenshots and data visualizations of Experiment 2. Screenshots of the *reappear* condition (*a*) and the *disappear* condition (*b*) in the videos at 540, 1800, 3060 and 4860 ms (from left to right). (*c*) Time series plot showing the dogs’ median (continuous black lines) and mean horizontal gaze coordinates (± *s.e.*, dotted line and the grey shaded area around it; in pixels (px)) in the final familiarization trials and in the test trials. The shaded yellow area represents the moving ball. The blue shaded areas indicate the location of the two screens. (*d*) Box plot showing the dogs' looking times to the areas of interest around the end position of the ball at the end of the video in the first test trial. The dots represent the mean individual values. (*e*) Time series plot showing dogs’ pupil sizes (in arbitrary units and baseline corrected). The blue and orange lines show the mean pupil size (± *s.e.*, shaded area around the line) in the *disappear* and *reappear* conditions, respectively. The dotted vertical lines show the time when the ball was occluded behind the left screen, the dash-dotted vertical lines the time period the ball was occluded behind the right screen. (*f*) Difference curve derived from GAMM02. The dashed line shows the estimated difference between the *disappear* and *reappear* test conditions; the shaded area shows the pointwise 95% confidence interval. The period during which the conditions differ significantly is highlighted in red.

In Experiment 2, the familiarization video showed a yellow-black patterned ball rolling along a grey surface from left to right. In the test videos, two blue walls were added to the scene (equidistant from the centre of the screen) placed in front of the path of the ball. There was a gap in between the two walls. The yellow-black patterned ball moved with exactly the same kinematics and along the same trajectory as in the familiarization. In the *reappear* condition (figure 2*a*), the ball disappeared behind the first (left) wall, reappeared between the two walls, disappeared behind the second (right) wall and finally reappeared on the right side of the second wall. In the *disappear* condition (figure 2*b*), the only difference was that the ball did not reappear in the gap between the two walls, i.e. the ball disappeared behind the left wall and reappeared from the right wall. The timings of the initial disappearing event and final reappearing event were the same across the test conditions.

#### (iii) Apparatus

We used the EyeLink1000 eye-tracking system (SR Research, Canada) to record the dogs’ eye movements and pupil sizes at 1000 Hz. We used an adjustable chin rest to facilitate the maintenance of a stable head position during stimulus presentation. We presented the stimuli on a 24-inch LCD monitor (resolution: 1024 × 768; refresh rate: 100 Hz) at a distance of 70 cm from the dogs' eyes. The video area subtended visual angles of 31.89 (horizontal) and 24.19 (vertical) degrees. The depicted ball had a diameter of *ca* 110 px (Experiment 1), 100 px (Experiment 2) subtending a visual angle of 3.47 and 3.16 degrees, respectively. We adjusted the height of the chin rest and the height and angle of the eye tracker for each subject. We trained the subjects prior to the study to keep their heads on the chin rest for up to one minute while the experimenter left the room and they had to pass a 5-point calibration and validation with an average deviation of a maximum of 1° of visual angle before entering the test.

#### (iv) Design and procedure

Experiments 1 and 2 used a within-subject design, in which we presented the subjects with two test conditions: the *disappear* condition, violating the physical principle under investigation, and the *reappear* condition, controlling for the novelty of the stimulus without violating this physical principle. The order of conditions was counterbalanced across subjects. We pseudo-randomly assigned the dogs to the order groups and counterbalanced the groups as much as possible with respect to age, sex and breed. We conducted two sessions per dog and experiment. Each session consisted of three identical familiarization trials followed by one (Exp. 1) or two (Exp. 2) test trial(s). In each experiment, we administered only one or two trials per test condition and six familiarization trials.

In each session, the dogs first completed a 5-point calibration with animated calibration targets (24–64 px) subtending visual angles of 0.77–2.05 degrees depending on the used target stimulus. Following the calibration, we presented a central fixation target (Exp. 1: a white expanding circle; max. diameter: 90 px; visual angle: 2.88 degrees; Exp. 2: the calibration target). The video started once the dogs fixated the target for 50 ms.

#### (v) Analysis

For the area of interest (AoI) analysis, we analysed the interest period at the end of the video when the last frame of the video was shown for *ca* 10 s (Exp. 1) / 3.5 s (Exp. 2). We showed the last frame of the experiment for an extended period of time to allow for a dwell time analysis with the moving objects in their final position. In Experiment 1, the ball was not visible any more at the end of the video. Here, we analysed an AoI around the pole (w × h: 300 × 460 px). In Experiment 2, we defined an AoI around the end position of the ball (w × h: 250 × 300 px). We compared the dwell time in each AoI across conditions using two-tailed, paired-samples *t*-tests.

For the analysis of the pupil size data, we pre-processed the data as follows [40]: we checked for artefacts by visually inspecting the data of each test trial. We excluded samples 100 ms before and after detected blink events and applied a linear interpolation. Next, we conducted a subtractive baseline correction. We used the entire pre-event period (i.e. before the ball moved behind the pole or the first wall) of the video for the baseline correction. Finally, we sampled the data down to 10 Hz to reduce autocorrelation.

We fitted a generalized additive mixed model (GAMM) with Gaussian error structure to analyze the pre-processed pupil size data (in line with our previous pupil size analyses (see [18,38]) and following the recommendations by [41,42]). We analysed a 4 s interest period starting at the end of the baseline period following the analysis pipeline of our previous expectancy violation study using pupillometry [18].

We have implemented the GAMM in R using the function ‘bam’ of package ‘mgcv’ [43] and package ‘itsadug’ [45] for visualization. We used smoothing parameter selection method ‘ML’. We included condition as a linear term, the nonlinear regression lines for time and for the two levels of condition over time (with the upper limit for the number of basis functions set to 20), and the nonlinear interaction between X and Y gaze positions (given that the gaze position can affect the pupil size) [40,42]. We also included random factor smooths for each subject and for each individual time series trajectory (i.e. for each subject and test trial) to improve the model fit and to account for autocorrelation [41,42].

We evaluated the model fit by inspecting visualizations of correlations between the residuals and the lagged residuals, a QQ-plot of residuals as well as the residuals against the fitted values (using the functions ‘gam.check’ of package ‘mgcv’ [43] and ‘acf’ of package ‘stats’ [44]). This revealed no substantial autocorrelation at lag 1 (Exp.1: 0.05; Exp.2: 0.09). The residuals seemed to be normally distributed and there was no obvious pattern in the residuals plotted against the fitted values.

To evaluate the significance of condition on pupil size, we compared the full model to a reduce model excluding both the parametric and smooth terms of condition using a chi-squared test of ML scores (using the function compareML of R package ‘itsadug’) [45]. Additionally, we inspected the model summary and we visually inspected the estimates of the differences between the conditions (using the function plot_diff of R package ‘itsadug’) [45].

### (b) Results and discussion

#### (i) Experiment 1

In Experiment 1, we tracked the dogs' eye movements while they watched short videos depicting a rolling ball moving past a narrow pole (electronic supplementary material, video S1). The ball either reappeared on the other side of the pole (*reappear* condition; figure 1*a*) or disappeared when moving past the pole (*disappear* condition; figure 1*b*). In line with our predictions, dogs showed increased pupil sizes and longer looking times in response to the *disappear* test event compared to the *reappear* event.

The dogs looked consistently at the screen while the video was playing (proportion of on-screen dwell time: familiarization: mean ± *s.e.*: 0.91 ± 0.01, *reappear*: 0.91 ± 0.01, *disappear*: 0.94 ± 0.01) and they followed the movement of the ball closely with their gaze (figure 1*c*). Their on-screen dwell time did not differ between the first and last familiarization trials (*t*(13) = 1.23, *p* = 0.239), but they looked overall longer at the screen in the *disappear* condition than in the *reappear* condition (*t*(13) = −2.36, *p* = 0.035). The AoI analysis revealed that the dogs looked significantly longer at the pole AoI in the interest period at the end of the video (when the ball was no longer visible) in the *disappear* condition than in the *reappear* condition (*t*(13) = 2.37, *p* = 0.034; figure 1*d*).

We analysed the preprocessed, baseline-corrected pupil size data (figure 1*e*; for the raw pupil size data, see electronic supplementary material, figure S1) using a generalized additive mixed model (GAMM01). The full model including condition fitted the data significantly better than the null model (chi-squared test of ML scores: *χ*^2^(5) = 9.83, *p* = 0.001; GAMM01 had a lower AIC: ΔAIC 15.38). The pupil size varied significantly over time in the *disappear* condition (*F*(10.46, 12.65) = 2.95, *p* < 0.001) but not in the *reappear* condition (*F*(0, 0) = 0.04, *p* = 0.993). The difference curve revealed that the pupil size was significantly larger in the *disappear* condition than the *reappear* condition in the time window between 2487 and 3901 ms (figure 1*f*; for the model estimates and partial and summed effects see electronic supplementary material, table S1 and electronic supplementary material, figure S2), even though the parametric term of condition failed to reach significance (*t* = 1.69, *p* = 0.092).

#### (ii) Experiment 2

In Experiment 2, we again tracked the dogs’ eye movements while they watched short videos depicting a rolling ball that moved from left to right past two screens with a gap in between (electronic supplementary material, video S2), similar to the test scenarios used to study occlusion events in human infants [e.g. 22–24]. In both test conditions, the ball reappeared on the right side of the right screen; however, in the *reappear* condition the ball was visible in between the two screens (figure 2*a*) whereas in the *disappear* condition it failed to reappear in between (figure 2*b*). In line with our predictions, dogs showed increased pupil sizes and longer looking times in response to the *disappear* condition compared to the *reappear* condition.

The dogs looked consistently at the screen while the video was playing (proportion of on-screen dwell time: familiarization: mean ± *se*: 0.90 ± 0.01, *reappear*: 0.89 ± 0.02; *disappear*: 0.86 ± 0.03) and they followed the movement of the ball closely with their gaze (figure 2*c*). Their on-screen dwell time did not differ between the first and last familiarization trials (*t*(16) = −0.83, *p* = 0.418) or between test conditions (first test trial: *t*(16) = 0.33, *p* = 0.742; second test trial: *t*(16) = 0.85, *p* = 0.407). When comparing the dwell times to the ball end position in the first test trial of each condition in the interest period at the end of the video (when the ball was no longer moving) we found significantly longer dwell times in the *disappear* condition than in the *reappear* condition (*t*(16) = 2.64, *p* = 0.018; figure 2*d*). We did not find this difference in the second test trial per condition (*t*(16) = 0.57, *p* = 0.579).

For the pupil size analysis (figure 2*e*; for the raw pupil size data, see electronic supplementary material, figure S3), we fitted another GAMM (GAMM02) with the same structure as in Experiment 1. We compared this model to a null model without the parametric and nonparametric terms of condition. The model including condition fitted the data significantly better than the null model (chi-squared test of ML scores: *χ*^2^(5) = 37.91, *p* < 0.001; GAMM02 had a lower AIC: *Δ*AIC 101.24). The pupil size varied significantly over time in the *reappear* condition (*F*(12.89, 14.16) = 2.49, *p* = 0.001) but not in the *disappear* condition (*F*(7.15, 8.08) = 0.47, *p* = 0.879). The difference curve revealed that the dogs had significantly larger pupils in the *disappear* condition than the *reappear* condition in the time window between 3108 and 5007 ms (figure 2*f*; for the model estimates and partial and summed effects see electronic supplementary material, table S2 and electronic supplementary material, figure S4), which was also supported by a significant parametric effect of condition (*t* = 3.27, *p* = 0.001).

The results of the first two experiments are consistent: dogs reacted to implausible occlusion events with dilated pupils and longer looking times either to the area where they saw the stimulus disappearing (the pole in Exp.1) or to the ball that did not re-appear between the screens in Experiment 2 when it finally reappeared. In humans, the pupil dilation that is part of an orienting response tends to peak with a delay of 0.5–1 s [46]; in dogs evidence so far suggests that this delay tends to be a bit longer, at 1–2 s [18,19]. With this in mind, the time course of the pupil size suggests that the pupil dilation effect was elicited by the disappearing event in Experiment 1. In Experiment 2, the pupil started dilating in both conditions when the ball first disappeared behind the left occluder and then continued to dilate in the *disappear* condition until it finally reappeared on the right side of the right screen. In the *reappear* condition, the pupil constricted following the reappear event in between the two screens. One might argue that dogs' pupil dilation was merely driven by moving objects suddenly disappearing irrespective of whether it is a plausible or implausible event. However, the pupil size trajectory in the *reappear* condition of Experiment 2 speaks against this possibility: it does not appear to increase when the ball moves past and disappears behind the right screen. Additionally, the gaze location data suggest that the dogs expected the reappearance of the ball in between the two screens because they looked at the gap in the period when the ball should have been there even in the *disappear* condition (figure 2*c*). Finally, the looking time effect at the end of the video of Experiment 2 (figure 2*d*) suggests that dogs were indeed surprised by the implausible occlusion event in the *disappear* condition.

## 3. Experiment 3: from expectancy violation to exploration

In this preregistered experiment (see https://osf.io/mzc6y), we replicated Experiment 2 using real world demonstrations and (video coded) looking times (electronic supplementary material, video S3). More importantly, we investigated whether expectancy-violating events would lead to more exploration of the involved object. We presented the dogs first with a familiarization in which a ball moved across a small stage. Then the dogs watched a single test event using the two-screen setup of Experiment 2. The ball either reappeared in between the two screens (*reappear* condition) or it did not reappear in between (*disappear* condition). Following this demonstration, we allowed the dogs to freely explore and interact with the target object (a replica of the object used during the demonstration) and two distractor objects. We recorded the dogs' looking times during the demonstration as well as their object interaction times during the exploration phase. Based on the results of Experiment 2, we predicted that the dogs would look for longer at the setup when their expectations concerning occlusion events were violated (*disappear* condition) compared to the control event (*reappear* condition). Moreover, we predicted that such an expectancy violation (in the *disappear* condition) subsequently would lead to increased exploration behaviour (as compared to the *reappear* condition).

### (a) Material and methods

#### (i) Subjects

Based on a power simulation (see electronic supplementary material, text for details), our preregistered target sample size was 68 dogs. We have tested 84 dogs accounting for dropouts. We excluded dogs from the analysis if there was an apparatus malfunction (*N* = 3), experimenter mistake (*N* = 2), any other interference (e.g. the handler communicating with the dog; *N* = 7), or if the dogs did not watch the critical test event (*N* = 1) or did not approach or interact with *any* of the objects in the exploration phase at all (*N* = 8). Our final sample consisted of 63 pet dogs (53 pure-bred dogs of 33 different breeds, 10 mixed breeds; mean age ± s.d.: 61.4 ± 29.4, range: 15–130 months; 33 females, 30 males). We recruited only dogs older than 12 months; otherwise, the recruitment was opportunistic (no restriction with respect to breeds, etc.).

#### (ii) Apparatus

**Figure 3.**
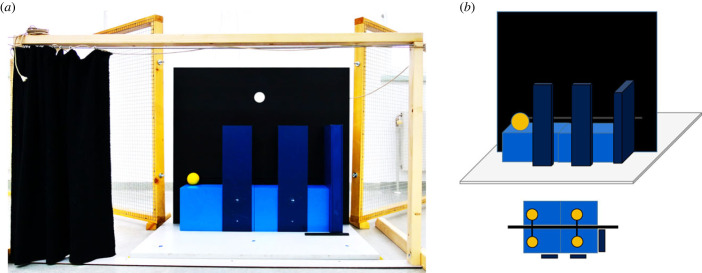
Setup of Experiment 3. (*a*) Photo; (*b*) Illustration (not to scale) showing the front view (top; i.e. the subjects' perspective) and bird's eye view of the *disappear* condition with two sets of balls (bottom).

The setup consisted of an apparatus resembling a small stage and a wooden frame (w × h: 150 cm × 83 cm; figure 3*a*) with a curtain that served to occlude the apparatus. Additionally, we used two 180 cm wide fence segments to fence off the dogs from the area behind the apparatus. The apparatus had a grey base plate (95 cm × 100 cm) and a black vertical plate in the centre (width × height: 90 cm × 80 cm). Two blue wooden boxes were in front of the vertical plate and a second set of identical boxes were behind the vertical plate (width × height × depth: 35 × 20 × 15 cm). The boxes were placed side-by-side to form a 70 cm-long surface on which the balls were moved during demonstrations. A yellow rubber ball (diameter: 6 cm) was placed on top of the boxes on the front side. Unseen by the dog, a metal rod protruded from the backside of the rubber ball. The metal rod extended through a horizontal slit (64 cm × 0.5 cm) in the vertical plate that formed the backside of the stage. On the backside of the stage, the rod was connected to a second identical ball that was placed on a second set of boxes. By sliding the ball on the backside, the experimenter (E) could make the visible ball on the front side move along the surface. A piece of black felt glued to the backside of the vertical plate along the entire width of the slit ensured that no light would shine through it and that the slit was hardly visible. In the familiarization and *reappear* test condition, we used one set of balls and in the *disappear* condition we used two sets of balls (figure 3*b*). On the right end of the two boxes (on the front side of the apparatus), another dark blue box (w × h × d: 5 cm × 50 cm × 20 cm) marked the endpoint of the ball's path. In the test condition, we mounted two dark blue partitions (w × h: 14 × 50 cm) on the front side of the boxes to occlude parts of the ball's path from the dogs' point of view. In between the two occluders, the path of the ball was visible (width of gap: 12 cm).

#### (iii) Design and procedure

In Experiment 3, we used a between-subject manipulation, i.e. we presented the subjects either with the disappear or reappear events of Experiment 2. The groups were counterbalanced as much as possible for age, sex and breed.

At the beginning of the session, the owner entered the testing room with the dog, sat on a chair facing the setup (occluded) and released the dog. Initially, there was a 2-min warm-up during which the experimenter (E) played with the dog with a rope toy (a toy that was not used in the experiment), showed the dog the curtain that would serve to occlude the apparatus during the experiment, and gave the dog some dry food. E showed the objects (which were presented in the exploration phase, i.e. the target and the distractor objects) to the dogs and let them sniff at them. Then E entered a fenced area inaccessible to the dog and sat behind the apparatus out of the dogs’ view and the test started.

In the initial demonstration phase, the dogs were presented with three familiarization trials followed by one test trial (either *disappear* or *reappear* condition). During this demonstration phase, the dog handler was blindfolded and instructed not to interact with the dog. At the beginning of each trial, the curtain occluding the apparatus opened (via a string controlled from behind the apparatus) and E called the dog's name to direct their attention toward the setup. The curtain was closed again at the end of a trial. In the familiarization trials, the ball was initially located on the left side of the stage. The hand of E appeared from behind the apparatus and moved as if setting the ball into motion. At the same time, E started moving the ball (from behind the apparatus) to the right side. E moved the ball until it reached the vertical partition on the right side of the apparatus. Once the ball reached its end position, E waited 5 s before closing the curtain.

In the test trial, the ball also moved from left to right (in the same manner as in the familiarization trials) but now two screens partly occluded the path of the ball. In the *disappear* condition, a second ball of the same appearance was hidden behind the right occluder. After the ball moved behind the left occluder, E moved the second, hidden ball after a short lag (similar to the duration it took the ball to roll from the left occluder to the right occluder) toward the end position on the right side of the right occluder. Thus, in the *disappear* condition it seemed as if the ball was not visible in the gap between the two occluders. In the *reappear* condition, we used only one ball, which we moved as in the familiarization. The ball was therefore visible in between the two occluders. In both conditions, E waited 20 s after the ball reached its end position before closing the curtain. In both conditions, there was a subtle sliding noise when the ball was moved that might have increased the salience of the demonstrations.

After the demonstration phase the exploration phase started. E removed the balls from the setup and placed the target and two distractor objects equidistant from dog's position in front of the apparatus. The distractor objects were two different dog toys: a yellow rubber ring and a blue dumbbell (electronic supplementary material, figure S5). We added two distractor objects to reduce the risk of ceiling effects (i.e. that all dogs would show the same preference for a particular object, which would have prevented us from analysing differences between conditions). The positions of the three objects were randomized across dogs. During the exploration phase, the handler was allowed to take off the blindfold but was instructed (prior to the experiment) not to interact with the dog. When the objects were in place, E opened the curtain (from behind the stage out of the dog's view) and the dog was allowed to freely explore and interact with the objects for three minutes.

#### (iv) Scoring and analysis

We scored the following response variables: the looking times to the setup during the demonstration phase, the durations the dogs interacted with each object (target or distractor) in the exploration phase, which object the dogs approached first (target or distractor), and whether or not the dogs approached the target object (yes / no). We scored an interaction with an object when the dog touched or sniffed an object. For exploratory analyses, we also scored how often the dogs pawed or dropped the target object in the exploration phase because we had observed these behaviours to be relatively frequent during piloting. Based on the object interaction times, we calculated two proportions: first, the proportion time the dogs interacted with the target object relative to the duration of the exploration phase (i.e. target interaction duration divided by duration of exploration phase) and, second, the proportion time the dogs interacted with the target objects compared to the other objects (i.e. target interaction duration divided by the total object interaction times).

A second coder naive to the experimental hypotheses scored 22% (*N* = 14) of the subjects. Inter-observer reliability was found to be good for all measures except for the number of pawing behaviours (intraclass correlation ICC1 [47]: looking times: *κ* = 0.68, *p* < 0.001; target interaction time: *κ* = 1.0, *p* < 0.001; distractor interaction time: *κ* = 0.97, *p* < 0.001; number of pawing events: *κ* = 0.55, *p* = 0.015; number of dropping events: *κ* = 0.92, *p* < 0.001; first approach: Cohens' *κ*: 0.85). Therefore, we did not further analyze pawing events.

Following our preregistered analysis plan (osf.io/mzc6y), we analysed the looking times by fitting a linear model with Gaussian error structure (using the R function lm [44]). We included condition (reappear, disappear) as a test predictor (factor), and age (in months) and sex as control predictor variables. The continuous predictor age was z-transformed to a mean of 0 and a standard deviation of 1. We detected no obvious violations of the assumptions (homogeneous and normally distributed residuals). We also looked at diagnostics of model stability (Cook's distance, DFBetas, DFFits, leverage), which revealed no obvious influential cases.

For the proportion interaction times, we fitted GLMs with β error structure using R function betareg [48]. We transformed the proportion data so that they did not comprise the extreme values 0 and 1 because these values cannot be modelled in β regressions [49]. For the binary dependent variables (target approached: yes/no; first approached object: target/distractor), we fitted binomial GLMs (logit link function) using R function glm. For the dropping behaviours (count response), we fitted a negative-binomial GLM using R function glm.nb [50]. We included the same predictor variables as before (condition, age, sex).

In case of the GLMs, inferences with respect to the fixed effects were drawn by performing likelihood ratio tests between the full model and reduced models lacking single predictor variables. For the GLMs with beta or negative-binomial error structure we also checked overdispersion which was no issue (dispersion parameter: target interaction relative to complete object interaction times: 1.11; target interaction relative to exploration phase: 1.13; dropping behaviours: 0.87). Finally, we also checked for collinearity, which was no issue in any of the models (variance inflation factor: 1.0).

### (b) Results

**Figure 4.**
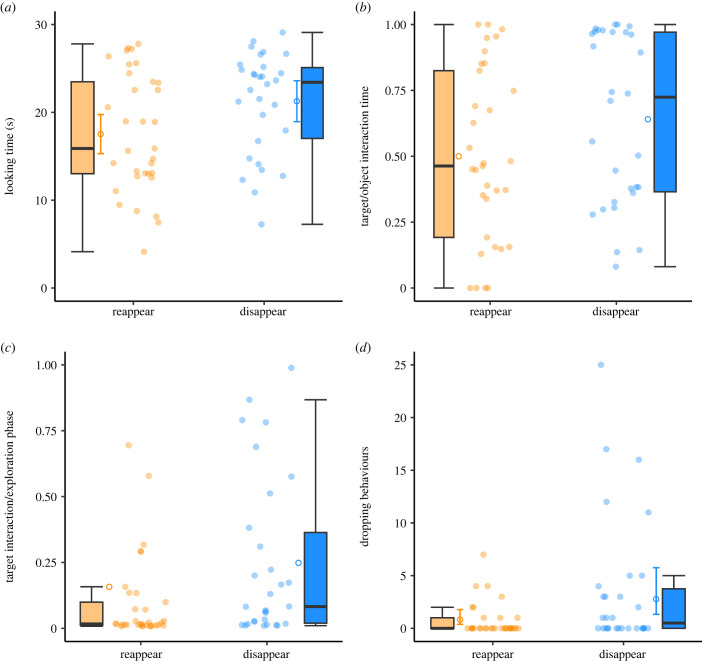
Data visualizations of Experiment 3. (*a*) Looking times at the setup during the demonstration phase (probe trial); (*b*) proportion target interaction times relative to entire object interaction times (target and distractors) in the exploration phase (object interaction proportion); (*c*) proportion target interaction times relative to the duration of the exploration phase (exploration phase proportion)*,* i.e. the target interaction times have been divided by the exploration phase duration (*ca* 3 min); (*d*) number of target dropping behaviours in the exploration phase. The filled circles represent the mean individual values. The open circles show the model fit. Error bars around the model fit represent 95% confidence intervals.

In line with our predictions, the dogs looked for significantly longer at the setup in the *disappear* condition than in the *reappear* condition in the probe trial of the demonstration phase (*t* = 2.32, *p* = 0.024; figure 4*a*). Sex or age had no significant effect on the looking times (see electronic supplementary material, table S3).

In the exploration phase, the dogs interacted with the target in the *disappear* condition on average for 43.9 s (SE: ± 10.3 s) and 16.4 s (± 5.4 s) in the *reappear* condition. We analysed the target interaction times as proportion relative to the complete object interaction times (object interaction proportion) and relative to the duration of the exploration phase (exploration phase proportion). For the object interaction proportion, we found that the dogs tended to interact more with the target object than with the distractors following the *disappear* event than the *reappear* event, although the effect was only marginally significant (*χ*^2^(1) = 3.61, *p* = 0.057; figure 4*b*). When analysing the exploration phase proportion, the dogs interacted with the target object for significantly longer following the *disappear* demonstration than the *reappear* demonstration (*χ*^2^(1) = 4.23, *p* = 0.040; figure 4*c*). Sex or age had no significant effect on the target interaction times (see electronic supplementary material, tables S4 and S5). Note that a potential general preference for the target object cannot account for the observed differences between conditions because we used the same (target and distractor) objects across conditions.

Most dogs interacted with the target object except for four dogs that were all in the *reappear* control group. Due to complete separation issues, we could not fit a binomial GLM on the binary target approached (yes/no) response variable; a Fisher's exact test provided no evidence for significant difference between conditions (*p* = 0.114). When analysing the first object that the dogs approached (binary response: target versus distractor) we found no evidence that the dogs' likelihood to approach the target object first was higher in the *disappear* than the *reappear* condition (*χ*^2^(1) = 0.14, *p* = 0.704; electronic supplementary material, table S6). In an exploratory analysis, we analysed specific exploratory behaviour, the dropping of the target to the ground. The dogs dropped the target behaviour more often in the *disappear* condition than the *reappear* condition (*χ*^2^(1) = 4.39, *p* = 0.036; figure 4*d* and electronic supplementary material, table S7). However, this result might be explained by the difference in target interaction times between conditions. Indeed, the effect of condition was no longer significant when we included only dogs that did interact with the target in the exploration phase (*N* = 59) and controlled for the target interaction time by including the log-transformed target interaction time as an offset term (*χ*^2^(1) = 2.39, *p* = 0.122).

## 4. General discussion

We examined across three experiments whether dogs preferentially oriented to events violating principles of occlusion and continuity. In line with our hypothesis, we found throughout the three experiments that dogs looked for longer at the expectancy-inconsistent event than the consistent one. Experiments 1 and 2 showed that dogs looked for longer at the moving stimulus following the implausible occlusion event. These interest-area-specific looking time analyses were further supported by pupil size analyses: dogs showed a more pronounced pupil dilation in response to the inconsistent event than to the consistent one. As shown in Experiment 3, this effect was not limited to the screen-based stimulus presentation and it even translated into action: following the expectancy-inconsistent *disappear* event, dogs explored the target objects for longer than following the expectancy-consistent *reappear* condition.

Dogs’ pupil dilation response to the *disappear* condition in Experiments 1 and 2 provides evidence that they were surprised by the sudden disappearance of the ball behind the pole (Exp. 1) and by its failure to reappear in between the two screens (Exp. 2). We observed this a pupil dilation response even though the ball in the familiarization trials (Exp. 1) also disappeared at the same location (but in contrast to the test events behind a wider screen). This finding matches previous work on dogs' knowledge of occlusion events: dogs reacted with increased smelling behaviour when a food item hidden inside a container was ‘magically’ replaced by a different food item while out of sight [28] and they looked for longer when the occluded object changed its size or colour while being occluded [17,29]. In another expectancy violation study, dogs that had witnessed a toy or reward being placed behind a screen looked for longer when the screen rotated as if no reward was present behind it [30]. Together, the previous studies suggested that dogs remember some properties of the hidden item and expect that the hidden object will constrain the movements of the overlying screen. The current study suggests that dogs also expect objects to reappear when they are no longer behind an occluding screen and this is consistent with the notion that they consider the size of the occluding object in relation the occluded object. However, further research is required to elucidate the specific variables that dogs use to discriminate between plausible and implausible occlusion events (following the developmental literature [22–24]), for example, by presenting scenarios in which the object is either taller or shorter than the screen [23].

Experiment 3 showed that expectancy violations enhance the exploration of the involved target object, similar to findings with human infants [3,5], despite large variation among individuals (possibly related to individual differences in playfulness, other personality traits and varying object preferences). This finding does not provide sufficient evidence that the dogs engaged in hypothesis testing (as argued for human infants [3]), that is, that they acted with the goal of examining the surprising property of the object that did not disappear when it should have done. A plausible interpretation of the current finding is that the increased attention that the dogs allocated to the target object following the *disappear* event carried over into the exploration phase without any particular goal-directedness in their exploration related to the expectancy violation. However, the finding that the dogs did not preferentially approach the target object first following the *disappear* event compared to the *reappear* event speaks against such a carry-over effect. More research will be needed to clarify whether dogs' exploration triggered by expectancy violations is goal-directed.

Human infants appear to tailor their exploration to the specific expectancy-violating event they observed [3], that is, they dropped the object more following an implausible support event (showing an unsupported object hovering through the air) and they pounded objects more against the ground following an implausible solidity event (in which an object appeared to move as if it moved through a solid wall). This finding has been interpreted as evidence for hypothesis-testing ability in preverbal infants. Our study (Exp. 3) was not designed to investigate links between the *type* of expectancy violation and *specific* exploratory actions. Future research should address the specificity of this link in dogs but also other animals, particularly in those that are known for their curiosity, object exploration, and tool use as they might be the most promising candidates in this respect [7].

To further advance our understanding of dogs' knowledge of occlusion events, a promising direction for future research will be to conduct a systematic investigation of the variables that may impact and potentially constrain it. For example, 2.5-month old infants are surprised when an object does not reappear between two screens but they are not surprised if instead of two screens one screen is used with a discontinuous lower edge, i.e. an inverted u-shaped screen [22,24]. Only at about 3 months of age can infants integrate information about lower edge discontinuity in their expectations about whether an object should be visible or not; from about 3.5 months of age, they integrate information about the height of the occluded object in relation to the size and shape of the screen [23,24]. Future research could examine whether the same variables (e.g. concerning the shape and size of the occluder) also affect dogs' reactions to occlusion events by manipulating the shape of the screens and the size of the occluded objects.

In conclusion, the current series of experiments provide evidence that dogs possess expectations regarding object visibility and occlusion. Dogs seemed to take into account the size of the occluded object in relation to the occluding screen, but additional research is needed to confirm this notion. We found that dogs' surprise response resulted in increased exploration of the involved object, which created opportunities for learning about the unusual properties of the object. However, it remains uncertain whether dogs explored the target object with the intention of filling gaps in their knowledge, and further investigation is necessary to clarify this point. Lastly, the method employed in our study shows promise for testing hypothesis-testing abilities not only in dogs but also in a wide range of other species.

## Data Availability

All data, code and materials used in the analyses are available on a public repository (doi:10.5281/zenodo.8013139) [51]. Supplementary material is available online [52].
